# Zoonotic enteric parasites transmitted from dogs in Egypt with special concern to *Toxocara canis* infection

**DOI:** 10.14202/vetworld.2015.946-957

**Published:** 2015-08-07

**Authors:** Maysa A. I. Awadallah, Lobna M. A. Salem

**Affiliations:** 1 Department of Zoonoses, Faculty of Veterinary Medicine, Zagazig University, Zagazig 44511, Egypt; 2 Department of Zoonoses, Faculty of Veterinary Medicine, Benha University, Benha 13518, Egypt

**Keywords:** dogs, enteric parasites, humans, risk factors, zoonoses

## Abstract

**Aim:**

This work aimed to study the role played by dogs in transmitting zoonotic enteric parasites to humans in Egypt and to analyze the risk factors associated with the occurrence of such infection in dogs. Serodiagnosis of anti-*Toxocara* immunoglobulin G (IgG) antibodies among human beings as well as analyzing risk factors predispose to *Toxocara canis* infection in human beings are another objectives of this study.

**Materials and Methods:**

From June to December 2013, a total of 130 fecal samples from 4 dog populations (Military, nomadic and domiciled dogs from rural and high standard districts) and 150 stool samples of 6 occupational groups were examined for the presence of enteric parasitic infection. Moreover, 150 serum samples were collected from humans from whom stool samples were collected and examined for the presence of anti-*T. canis* antibodies.

**Results:**

Enteric parasites were detected in 30% of fecal samples from 4 dog populations in Egypt. High infectivity had been reported in nomadic dogs (63.33%) (Crude odds ratios [COR]=67.36, 95% confidence interval [CI]=8.09-560.8, p<0.000), followed by domiciled dogs from rural areas (40%) (COR=26, 95% CI=3.14-215.54, p=0.003), domiciled dogs from high standard areas (23.33%) (COR=11.87, 95% CI=1.37-102.69, p=0.025) and military dogs (2.5%). Twelve species of enteric parasites were identified, *Ancylostomatidae* (6.15%), *T. canis* and *Cryptosporidium* spp. (5.38%, each), *Heterophyes* spp. (3.85%), *Toxocara leonina* and *Blastocystis* spp. (3.07%), *Taenidae* eggs (2.31%), *Hymenolepis diminuta* (1.54%) and *Entamoeba canis*, *Cyclospora cayetanensis*, and *Paragonimus* spp. (0.77%, each). Univariate logestic regression revealed significant association of age (COR=4.73, 95% CI=2.13-10.53, p<0.000), gender (COR=2.63, 95% CI=1.22-5.68, p<0.014), housing system (COR=5.10, 95% CI=2.04-12.75), p<0.000) with enteric parasitic infection in dogs. However, breeds (COR=6.91, 95% CI=0.88-54.52, p=0.067) and type of feeding (COR ranged from 3.5 to 7.62, p>0.05) did not seem to have a significant association among the examined dogs. Enteric parasitic infection was reported in 31/150 human stools (20.67%). Students were the most affected groups (37.14%), followed by nomadic people (24%), house wives (20%), house guarders and military workers (12%, each), and employees (10%). The identified parasites were *Cryptosporidium* spp. (9.33%), *Ascaris lumbercoides* (3.33%), *Heterophyes* spp. and *Ancylostoma* spp. (2.66%, each) and *Paragonimus* spp. and *Hymenolepis nana* (1.33%, each). *Toxocara* IgG antibodies were detected in 36/150 (24%) serum samples investigated. Toxocara IgG antibodies were more prevalent in males (26.66%) than females (20%). Seroprevalence was highest (17/35, 48.57%) in 7-15 years old (COR=6.93, 95% CI=1.75-27.43, p=0.006). Seroprevalence values for *T. canis* antibodies were higher in those; raising dogs (29.85%), eating raw vegetables (25.21%) and not washing hands before meals (25.45%). *T. canis* antibodies were detected in 25% of those contacted with soil compared to 30% of those did not. Students were mostly affected (34.29%), followed by nomadic people (32%), house guarders (28%), housewives (20%), military workers (13%), and employees (10%).

**Conclusion:**

Detection of enteric parasites in dogs and humans in Egypt substantiates the role posed by dogs in transmitting zoonotic parasites to humans and knock an alarm for common sources of infection for humans and dogs. Common sources may be infected fish or contaminated vegetables that are consumed by dogs or humans or even infected rodents that may contaminate their feed. This pilot study necessitate the need for similar studies and tracing such infection in fish, vegetables, rodent that may be responsible for infecting humans and dogs in order to understand the epidemiology of zoonotic parasitic infection transmitted from dogs to humans.

## Introduction

“Dogs are the most successful canids, adapted to human habitation worldwide.” They provide their owners, particularly children with physical, social and emotional benefits [1]. However, in spite of these benefits, close contact of dogs with humans remain a major threat to public health as dogs are main reservoirs of many infective stages of parasites that can be transmitted to man and other domestic animals [1,2]. Dogs act as definitive or reservoir hosts for more than 60 zoonotic parasites, such as *Toxocara canis*, *Echinococcus* spp., *Taenia* spp., *Dipylidium caninum*, *Ancylostoma* spp., *Giardia* spp., as well as *Cryptosporidium* spp. [3].

In Egypt, like other developing countries, the risk of zoonotic infection related to domiciled, as well as stray dogs is high due to keeping of livestock and pets inside houses in most rural areas [4], less restrictive obligation placed on dog owners [5] and absence of public education about the risk of zoonotic diseases transmitted from dogs, as well as non-existence of a control strategy for stray dogs [6].

Toxocariasis is a zoonotic disease caused by *T. canis*. Human toxocariasis constitutes one of the most common parasitic infections worldwide, which is more prevalent in developing countries and poor communities. It is caused by ingesting the eggs which were shed in the feces of the definite dog host [7]. Human infection is mostly asymptomatic. Larvae liberate from the eggs in the intestines, penetrate the mucosa, escape to the portal circulation and disseminated into various organs where could possibly become encysted. *T. canis* cannot mature in humans due to inability to come back to the intestines where they normally go to in dogs, to lay eggs.

Demonstrating the presence of *T. canis* through traditional diagnostic methods is hard and has remained unsatisfactory as the parasite does not develop nor reproduce in man. Measuring anti-*Toxocara* immunoglobulin G (IgG) antibodies to excretory–secretory antigens of the larval stage of *T. canis* using Enzyme linked immunosorbent assays (ELISA) are the best laboratory option for diagnosis [7].

Zoonoses involving dog parasites are both common and important, with some causing serious diseases. Understanding the epidemiology of zoonotic parasitic infections is important to minimize the risk of human infection. The aim of this work was to study the role played by dogs in transmitting zoonotic enteric parasites to humans and to analyze the risk factors associated with occurrence of such infection in dogs. Information on the epidemiology of human toxocariasis in Egypt is scarce so serodiagnosis of anti-*Toxocara* IgG antibodies among human beings, as well as analyzing risk factors predispose to *T. canis* infection in human beings are another objectives of this study.

## Materials and Methods

From June to December 2013, a total of 130 fecal samples from 4 dog populations (Military, nomadic and domiciled dogs from rural and high standard districts) and 150 stool samples of 6 occupational groups were examined for the presence of enteric parasitic infection. Moreover, 150 serum samples were collected from humans from whom stool samples were collected and examined for the presence of anti-*T. canis* antibodies.

### Ethical approval

Ethical approval from dogs’ owner and assurance of anonymity, witnessed by a veterinarian from the Egyptian Veterinary Medicine Authority was obtained. Stool swabs and blood samples were also collected from human populations who gave an oral consent from the adult participants and parents of school children to participate in the present work.

### Sampling

#### Fecal samples of dogs

Samples from 60 domiciled dogs were collected by the authors during morning visits to 40 randomly chosen domiciles situated in a high class district at Al-Obour city, Qalyubia province and a rural district at Meet El-Ezz village, Fakous city, Sharkia province (30 dogs, from 20 home in each district). All these were semi-restricted dogs, housed indoors or in the yard, reared without free access to the street. Samples from nomadic dogs (n=30) were obtained by their owners at different localities at Sharkia and Qalyubia provinces. These dogs were free roaming, housed outdoors, fed on garbage or were allowed to scavenge on rodents and dead animals and chickens. However, samples from military dogs (n=40) were collected by their care takers. These dogs were highly confined and received high level of care and veterinary attention. All animals did not receive any kind of medicine 4 weeks before collection of samples. Fresh fecal samples were collected from each dog population by the responsible person mentioned above from the ground in the morning after voiding by dogs and individually labeled in plastic container. Number of each dog population was illustrated in Table-1. Each sample was preserved in formalin (10%) until examined.

**Table-1:** Frequency of enteric parasites in four dog populations from different localities in Egypt.

Parasite spp.	Populations of dogs (positive (%))				Total (n=130)

	Military dogs (n=40)	Domiciled dogs from		Nomadic (n=30)	

		High standard area (n=30)	Rural area (n=30)		
*Ancylostoma* spp.	1 (2.5)	1 (3.33)	2 (6.66)	4 (13.3)	8 (6.15)
*T. canis*	-	1 (3.33)	2 (6.66)	4 (13.3)	7 (5.38)
*T. leonine*	-	-	1 (3.33)	3 (10)	4 (3.07)
*D. caninum*	-	-	-	2 (6.66)	2 (1.54)
*Cryptosporidium* spp.	-	1 (3.33)	2 (6.66)	4 (13.33)	7 (5.38)
*Blastocystis* spp.	-	2 (6.66)	2 (6.66)	-	4 (3.07)
*E. canis*	-	1 (3.33)	-	-	1 (0.77)
*C. caytanensis*	-	-	-	1 (3.33)	1 (0.77)
*Paragonimus* spp.	-	-	1 (3.33)	-	1 (0.77)
*Heterophyes* spp.	-	-	4 (13.33)	1 (3.33)	5 (3.85)
*H. diminuta*	-	-	-	2 (6.66)	2 (1.54)
*Taenidae* eggs	-	2 (6.66)	-	1 (3.33)	3 (2.31)
Mono-infection	1 (2.5)	6 (20)	10 (33.33)	16 (53.33)	33 (25.38)
Mixed infection	-	1 (3.33)^a^	2 (6.66)^b^	3 (10)^c^	6 (4.6)
Total parasite infection	1 (2.5)	7 (23.33)	12 (40)	19 (63.33)	39 (30)

a Mixed infection with *Cryptosporidium* spp. + *Blastocystis* spp.

b Mixed infection with *Cryptosporidium* spp. + *Blastocystis* spp. and *Heterophyes* spp. + *Blastocystis* spp.

c Mixed infection with *Cryptosporidium* spp. + *Ancylostoma* spp., *Cryptosporidium* spp. + *T. leonina* and *Cryptosporidium* spp. + *Cyclospora caytanensis*

#### Human stool samples

A total of 150 stool samples from 6 occupational groups were collected by distributing clean cups on them a day before sampling and informing them to collect morning stool. Then the author visited them to pick up the collected stools. Number of each occupational group was illustrated in Table 2.

**Table-2:** Frequency of enteric parasites in 6 occupational groups at different localities in Egypt.

Enteric parasite spp.	Occupational groups (positive (%))						Total (n=150)

	Military workers (n=25)	Nomadic people (n=25)	House guarders (n=25)	Employees (n=20)	House wives (n=20)	Students (n=35)	
*Heterophyes* spp.	-	1 (4)	-	-	1 (5)	2 (5.71)	4 (2.67)
*Paragonimus* spp.	-	1 (4)	-	1 (5)	-	-	2 (1.33)
*Cryptosoridium* spp.	2 (8)	1 (4)	2 (8)	1 (5)	1 (5)	7 (20)	14 (9.33)
*Ascaris lumbercoides*	1 (4)	2 (8)	-	-	1 (5)	1 (2.86)	5 (3.33)
*Ancylostoma* spp.	-	1 (4)	1 (4)	-	1 (5)	1 (2.86)	4 (2.67)
*Hymenolepis* nana	-	-	-	-	-	2 (5.71)	2 (1.33)
Total	3 (12)	6 (24)	3 (12)	2 (10)	4 (20)	13 (37.14)	31 (20.67)

### Parasitological examination

Each fecal sample was examined macroscopically for adult nematodes and tapeworm proglottids. Each sample was subjected for examination by centrifugal fecal floatation technique using zinc sulphate solution [8]. Furthermore, formol ether sedimentation technique [9] was applied for each sample. Iodine solution was used to facilitate protozoan and cyst identification. The modified Ziehl-Neelsen staining technique is used to detect oocysts in the feces. Parasites were identified on the basis of eggs, oocysts or cysts color, shape, and contents [8].

### Serodiagnosis of anti-*T. canis* antibodies in human sera

A total of 150 serum samples were collected from participant from whom stool samples were collected and subjected to ELISA for detection of anti-*T. canis* antibodies using RIDASCREEN^®^ *Toxocara* IgG Kits (K7421) as the followings:

Microwell plates coated with *T. canis* antigen were inoculated with 100 μl diluted sera in buffer diluent (1:50) and ready-to use negative and positive controls and incubated at 20-25°C for 15 min. The wells were then emptied into a waste container containing any disinfectant. After that, the plates were knocked out onto absorbent paper in order to remove the residual moisture. The plates were washed 5 times using 300 μl of diluted washing buffer (1 part wash buffer “phosphate-buffered NaCl solution” concentrate is mixed with 19 parts distilled water) each time. The wells were emptied completely by knocking them out on absorbent paper after each wash. One hundred microliter of the conjugate was added to each well. The plates were then incubated at 20-25°C for 15 min and then washed 5 times. One drop or 50 μl of each of the substrate (urea peroxide) and chromogen (tetramethylbenzidine) were placed in each well and then incubated at 20-25°C for 15 min. The reaction was stopped by adding 50 μl (or 1 drop) stop reagent (0.5 M sulphuric acid) to each well. After mixing carefully (by lightly tapping the side of the plate), the absorbance was measured at 450 nm in a plate photometer. The results were then evaluated and interpreted as the followings:

- Calculating the sample index:
  1. The average absorbance is calculated for the negative control.
  2. The cut-off for the test was calculated by adding 0.150 to the average absorbance of the negative control.
  3. The sample index is obtained by dividing the absorbance for the sample by the cut-off.
- Interpreting the test as the followings
  Sample index <0.9 is considered negative, 0.9−1.1 is equivocal and >1.1 is positive.

### Risk factors assessment

The potential risk factors substantiate the occurrence and maintenance of enteric parasitic infection among the examined dog populations were studied through a questionnaire designed to obtain information about number, age, gender, breed, feeding, and housing system of each population (Table-3). Moreover, age and gender distribution of anti-*T. canis* antibodies as well as the influence of some risk factors on seroprevalence of *T. canis* infection in human populations were studied through a questionnaire included age, gender, raising of dogs, contact with soil, eating raw vegetables, washing hands before eating, as well as the occupation of the examined human populations (Table-4).

**Table-3:** Risk factors for enteric parasitic infection in the investigated dog populations.

Risk factors	Total examined	Enteric parasitic infection (n (%))		COR	p value

		−ve	+ve		
Age					
>1 year	83	68 (81.90)	15 (18.10)	1	<0.000
<1 year	47	23 (48.90)	24 (51.10)	4.73 (2.13-10.53)	
Gender					
Male	78	61 (78.20)	17 (21.80)	1	0.014
Female	52	30 (57.70)	22 (42.30)	2.63 (1.22-5.68)	
Breed					
Exotic	15	14 (93.30)	1 (6.70)	1	0.067
Local	115	77 (67.00)	38 (33.00)	6.91 (0.88-54.52)	
Housing					
Individual	55	48 (87.30)	7 (12.70)	1	<0.000
Communal	75	43 (57.30)	32 (42.70)	5.10 (2.04-12.75)	
Dog population					
Military dogs	40	39 (97.50)	1 (2.50)	1	<0.000
High standard area domiciled dogs	30	23 (76.70)	7 (23.30)	11.87 (1.37-102.69)	0.025
Rural areas domiciled dogs	30	18 (60.00)	12 (40.00)	26.00 (3.14-215.54)	0.003
Nomadic dogs	30	11 (36.70)	19 (63.30)	67.36 (8.09-560.81)	<0.000
Feeding					
Dry feed	15	14 (93.30)	1 (6.70)	1	0.108
Uncooked	105	68 (64.80)	36 (34.28)	7.62 (0.96-60.24)	0.054
Cooked	10	8 (80.00)	2 (20.00)	3.50 (0.27-44.95)	0.336

COR=Crude odd ratio

**Table-4:** Demographic characteristics of the seroprevalence of *T. canis* IgG antibodies among various human populations in Egypt.

Risk factor	Groups	Number of examined	Number of positive (%)	COR (95% CI)	p value
Gender	Male	90	24 (26.66)	1.46 (0.66-3.19)	0.35
	Female	60	12 (20)	1	
Age in years	7-15	35	17 (48.57)	6.93 (1.75-27.43)	0.006*
	16-25	46	8 (17.39)	1.54 (0.37-6.43)	0.551
	26-35	44	8 (18.18)	1.63 (0.39-6.80)	0.503
	36-45	25	3 (12)	1	0.003*
Raising dogs	Yes	67	20 (29.85)	1.78 (0.84-3.79)	0.134
	No	83	16 (19.27)	1	
Contact with soil	Yes	120	30 (25)	1.33 (0.50-3.57)	0.567
	No	30	6 (30)	1	
Eating raw vegetables	Yes	115	29 (25.21)	1.35 (0.53-3.42)	0.528
	No	35	7 (20)	1	
Washing hands before eating	Yes	40	8 (20)	0.73 (0.30-1.78)	0.49
	No	110	28 (25.45)	1	
Occupation	Military workers	25	3 (13)	1	0.231
	Nomadic people	25	8 (32)	3.45 (0.79-15.01)	0.099
	House guarders	25	7 (28)	2.85 (0.64-12.64)	0.168
	Employees	20	2 (10)	0.82 (0.12-5.42)	0.832
	House wives	20	4 (20)	1.83 (0.36-9.35)	0.466
	Student	35	12 (34.29)	3.83 (0.95-15.42)	0.059

COR=Crude odd ratio, CI=Confidence interval, IgG=Immunoglobulin G, *T. canis=Toxocara canis,*

* =Significant association

### Statistical analysis

Univariate logistic regression models were fitted to determine risk factors associated with prevalence of enteric parasite spp. in 4 populations of dogs from different localities in Egypt and to study the association of some risk factors with seropositivity of *T. canis* antibodies among 6 occupational groups in Egypt. The statistical software packages (SPSS for Windows 21.0, Inc., Chicago, IL, USA) was used for data ­analysis and results are expressed as numbers and percentages in brackets along with crude odds ratios (COR) and their 95% confidence interval (95% CI).

## Results

### Occurrence of enteric parasitic infection in dogs

**Figure-1:**
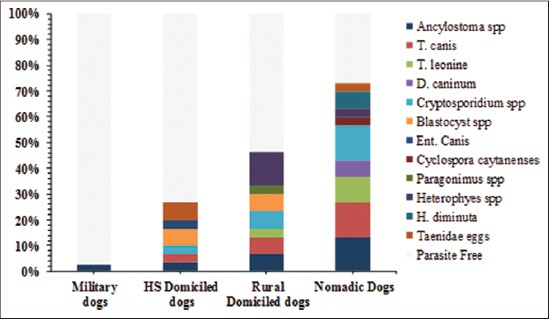
Frequency of enteric parasites in four dog populations from different localities in Egypt. HS=High standard domiciled dogs.

**Figure-2:**
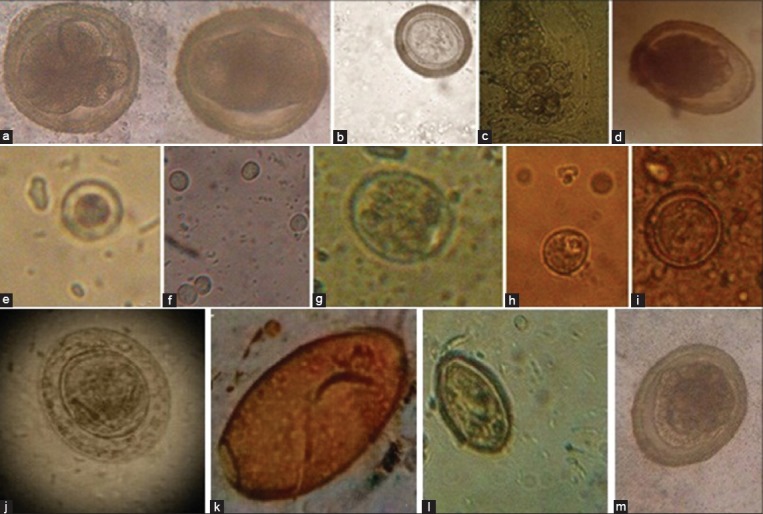
The identified enteric parasites. (a) *Ancylostoma* eggs, (b) *Taenia* eggs, (c) *Dipylidium caninum* eggs, (d) *Ascaris lumbercoides* eggs, (e) *Blastocystis hominis*, (f) *Cryptosporidium* oocyst, (g) *Cyclospora caytanesis*, (h) *Entamobea canis*, (i) Toxocara *leonina*, (j) *Hymenolepis nana*, (k) *Paragonimus westermani*, (l) *Heterophyes* eggs, (m) *Toxocara canis* eggs.

The overall frequency of enteric parasitic infection of dogs was 30% (39/130), Comparing the prevalence of infection among the four dog populations examined, high infectivity had been reported in nomadic dogs (63.33%), followed by domiciled dogs from rural areas (40%), domiciled dogs from high standard areas (23.33%), however, military dogs had the lowest prevalence (2.5%) of parasitic infection (Table-1). Twelve species of intestinal enteric parasites were detected, in particular *Ancylostomatidae* (6.15%), *T. canis* and *Cryptosporidium* spp. (5.38%, each), *Heterophyes* spp. (3.85%), *Toxocara leonina* and *Blastocystis* spp. (3.07%, each), *Taenidae* eggs (2.31%), *Hymenolepis diminuta* and *Dipylidium caninum* (1.54%, each) and *Entamoeba canis*, *Cyclospora caytanensis*, and *Paragonimus* spp. (0.77%, each). The majority of dogs were infected only with one species parasite (25.38%). Mixed infection caused by two species was recorded in 4.6% of all examined dogs (3.33% in domiciled dogs from high standard areas, 6.66% in domiciled dogs from rural areas and 10% of nomadic dogs) (Table-1, Figure-1 and Figure-2).

### Risk factors assessment for maintenance of enteric parasitic infections among the four dog populations

Risk factors assessment for maintenance of enteric parasitic infections among the examined dogs using univariate logistic regression models revealed significant association of age (COR=4.73, 95% CI=2.13-10.53, p<0.000), gender (COR=2.63, 95% CI=1.22-5.68, p<0.014), housing system (COR=5.10, 95% CI=2.04-12.75), p<0.000) with the frequency of enteric parasitic infection among the examined dogs. However, breeds (COR=6.91, 95% CI=0.88-54.52, p=0.067) and type of feeding (COR ranged from 3.5 to 7.62, p>0.05) did not seem to have a significant association. Univariate logistic regression revealed that nomadic dogs were 67.36% more likely to be infected with enteric parasites than military dogs (95% CI=8.09-560.8, p<0.000) followed in descending order by dogs from rural areas (COR=26, 95% CI=3.14-215.54, p=0.003) and dogs from high standard areas (COR=11.87, 95% CI=1.37-102.69, p=0.025) (Table-3).

### Occurrence of enteric parasitic infection in humans

Of 150 stool samples collected from 6 occupational groups and examined for the presence of enteric parasitic infection, 31 samples (20.67%) were positive. Students were the most affected groups (37.14%), followed by nomadic people (24%), house wives (20%), house guarders and military workers (12%, each), and employees (10%). Six parasite species were detected. *Cryptosporidium* spp. was the most frequently detected species (9.33%), followed by *Ascaris lumbercoides* (3.33%), *Heterophyes* spp. and *Ancylostoma* spp. (2.66%, each) and *Paragonimus* spp. and *Hymenolepis nana* (1.33%, each) (Table-2 and Figure-2).

### Occurrence of *Toxocara* IgG antibodies in humans and risk factor assessment

Of 150 serum samples investigated, 36 (24%) were positive for *Toxocara* IgG antibodies as determined by RIDASCREEN^®^ Toxocara IgG ELISA. *Toxocara* IgG antibodies were more frequent in males (26.66%) than females (20%). Seroprevalence was highest (17/35, 48.57%) in 7-15 years old, followed in sequence by 18.18% (8/44) in 26-35 years old, 17.39% (8/46) in 16-26 years old and 12% (3/25) in 35-50 years old. Seroprevalence values for *T. canis* antibodies were higher in those; raising dogs (29.85%, 20/67), eating raw vegetables (25.21%, 29/115) and not washing hands before meals (25.45%, 28/110). *T. canis* antibodies were detected in 25% of those contacted with soil compared to 30% of those did not. Students were mostly affected (34.29%), followed by nomadic people (32%), house guarders (28%), housewives (20%), military workers (13%), and employees (10%) (Table-4). Univariate logistic regression analysis revealed only a significant association of age of the examined humans with the seropositivity for *T. canis* antibodies (COR=6.93, 95% CI=1.75-27.43, p=0.006) among 7-15 years old age group (Table-4).

## Discussion

### Occurrence of enteric parasitic infection in dogs

“In many parts of the world, the intestinal parasites of dogs receive considerable attention because dogs serve as reservoirs, carriers and transmitters of several pathogens, including parasites, which are considered zoonotic and a number of them are of significant public health concern” [10]. The overall frequency of enteric parasitic infection of dogs in this study revealed a high level of infection (39/130, 30%) compared to the finding (33/180, 18.3%) from recent study carried out in northern part of Egypt [11]. The difference between both studies in spite of conducting at the same country may be related to difference in locality and nature of the examined dogs. They collected their samples from Alexandria province that has higher socio-economic level than Sharkia and Qalyubia provinces from which our samples have been collected. Moreover, their study was confined to 120 police dogs and 60 house dogs that receive high level of care (hygienic measures and veterinary attention) than nomadic and rural dogs included in the current study. Higher prevalence of enteric parasitic infection among the examined dogs; 76% in South Africa [12], 85% in Mexico [13], 71% in Spain [14], 68.4% in Nigeria [15], 39.2% in Japan [16] and 46.7% in Nepal [3] were previously recorded. “The wide range of endoparasite prevalence may be related to geographical location, status of animal ownership, sampling protocols, demographic factors, anthelmintic usage, and diagnostic techniques” [17].

Mono-infection was the rule during this study in all dog populations; however, mixed infection was reported in a domiciled dog from high standard areas, 2 domiciled dog from rural areas and 3 nomadic dogs. These results agreed with those reported by Katagiri and Oliveira-Sequeira [17] who stated that dogs harboring one parasite were more common (31.4%) than those harboring two (18.5%), three (3.2%) or four (1.2%) and Ugbomoiko *et al*. [15] who reported single infection in 49.4% of examined dogs compared to 13.1%, 4.5% and 1.3% for those harboring two, three and four or more parasites, respectively. On the other hand, mixed infection with more than one species was more common than mono-infection as reported by Dalimi *et al*. [18], (75.68% for polyinfection vs. 24.32% for mono-infection) and Eslami *et al*. [19] who found that 80% of the examined dogs were polyparasitised.

Twelve species of enteric parasites were detected in fecal samples of dogs, in particular *Ancylostomatidae* (6.15%), *T. canis* and *Cryptosporidium* spp. (5.38%, each), *Heterophyes* spp. (3.85%), *T. leonina* and *Blastocystis* spp. (3.07%), *Taenidae* eggs (2.31%), *H. diminuta* and *D. caninum* (1.54%, each) and *Entamoeba canis*, *Cyclospora cayetanensis*, and *Paragonimus* spp. (0.77%, each). The same parasite species were recorded in dogs all over the world. *Ancylostoma* spp., were previously recorded in dogs with percentages of 37.8 in Brazil [17], 16.9 in Nigeria [15] and 1.9 in Japan [16].

*T. canis* were frequently recorded in dogs either, stray, domiciled, police or military dogs. Ahmed *et al*. [11] in Egypt detected *T. canis* in 0.8% of police dogs and 5% of house dogs. In Iran, Eslami *et al*. [19], Mirzaei and Fooladi [20] and Adinezadeh *et al*. [21] detected *T. canis* in 22%, 7% and 4.3% of the examined stray dogs, respectively. Moreover, *T. canis* infections were recorded in dogs with values of, 1.4% of domiciled dogs in UK [22], 13.9% of stray dogs and 3.2% of domiciled dogs in Brazil [17], 41.7% of domiciled dogs in Nigeria [15], 13.3% of military dogs in Turkey [23] and 25% of stray dogs in Japan [16].

*T. leonina* was previously detected in house dogs (1.7%) but not in police dogs in Alexandria, Egypt [11]. Dalimi *et al*. [18], Adinezadeh *et al*. [21] and Kimura *et al*. [16] detected *T. leonina* in 32.53%, 53% and 0.5% of the examined stray dogs, respectively.

Similar frequency of *Cryptosporidium* spp. in faeces of dogs (5%) to those recorded in this study (5.38%) was previously reported by Fatahi-Burani [24]. Lower prevalence of *Cryptosporidium* spp. was previously recorded by Katagiri and Oliveira-Sequeira [17] in Brazil (3.1%) and Ahmed *et al*. [11] in Egypt (1.7%). Detection of *Cryptosporidium* spp. oocyst in the examined dogs may be attributed to the fact that dogs eat faces of dogs, as well as faces of many other species as well. “This brings the possibility that *Cryptosporidium* was from ingested faces of other species and may just have been passing through the dog, not an actual infection.” The lack of these infections in military dogs which were well managed and not allowed to roam would support this.

Nearly similar frequency of *D. caninum* to those recorded in this study were reported by Katagiri and Oliveira-Sequeira [17] and Eslami *et al*. [19] whose results were 4.6% and 4%, respectively. However, Adinezadeh *et al*. [21] detected *D. caninum* in 46% of the examined dogs. In Giza, Egypt [25] detected *D. caninum* in 13.33% of puppies and 66.67% of adult dogs immediately after hunting and in 53.33% of puppies and 66.6% of adult dogs after 3 months in captivity.

Blastocystis spp. was detected in 3.07% of the examined dogs in this study. Recently, a special concern has been paid to Blastocystis spp. as agent of human intestinal disease. A high prevalence of Blastocystis hominis has been reported in Egypt among 22.4% of asymptomatic patients [26] and 12.1% of patients with diarrhea and immunosuppressed children [27]. Blastocysis spp., were previously isolated from 10% of the examined cats in Sharkia province, Egypt [28]. *Blastocystis* spp. causes a variety of nonspecific symptoms including intense abdominal disorders, together with pain; diarrhea and constipation were reported in most cases [29].

*H. diminuta* was detected in 1.54% of the examined dogs in this study. El Shazly *et al*. [26] detected *H. diminuta* in 1.4% of human stools in Dakahlia province, Egypt. Moreover, Abd El-Wahed *et al*. [30] stated that rodents are considered the main reservoir of this parasite in Egypt that may infect animals or humans.

### Risk factors assessment for maintenance of enteric parasitic infections among the 4 dog populations

Potential risk factors for occurrence and maintenance of parasitic infection among various dog populations at different localities in Egypt were studied during this work (Table-3). This study showed a significantly higher enteric parasitic infection (p>0.000) in dogs under the age of 1 year (51.10%) compared with those above 1 year old (18.10%). Higher prevalence of enteric parasitic infection in puppies than adults were previously recorded by Eslami *et al*. [19] in Iran, Abere *et al*. [31] in Ethiopia and Ahmed *et al*. [11] in Egypt. They attributed this finding to the age acquired specific immunity against parasites or probably as a result of multiple re-infections. Moreover, Oliveira-Sequeira *et al*. [6] related the highest occurrence in puppies to transplacental or transmammary infection during the first few days of life.

Regarding gender as a risk factor for acquiring enteric parasitic infection, female dogs were 2.63 times at risk of acquiring enteric parasitic infection than male dogs (p=0.014). These results agreed with Davoust *et al*. [32] in Gabon and Ahmed *et al*. [11] in Egypt. On contrary, Zelalem and Mekonnen [33] in Ethiopia reported high prevalence of enteric parasitic infection in male dogs (79.2%) than female ones (76.8%). However, Mirzaei and Fooladi [20] and Gharekhani [34] reported non-significant difference in the overall prevalence between males and females.

Breeds were found to be a non-significant risk factor for occurrence of enteric parasitic infection among dog populations in Egypt (33% in local breeds versus 6.7% in exotic breeds, COR=6.91, 95% CI=0.88-54.52, p=0.067). Zelalem and Mekonnen [33] reported higher prevalence of helminthes in exotic breeds (81.3%) than local breed dogs (76.5%). However, Swai *et al*. [35] in Tanzania and Ahmed *et al*. [11] in Egypt stated that all breeds equally susceptible to infection if exposed to infected material. The higher occurrence of enteric parasitic infection among local than exotic breeds examined during this study may be related to the fact that Egyptians deal with exotic breeds with great care, vaccinate and deworm them, not let them move freely out doors and seek a veterinarian support if they acquired any illness due to their high prices.

Parasitic infection was found to be more frequent in dogs fed uncooked feeds (34.28%) than those fed cooked (20%) and dry (6.7%) feeds. Univariate regression analysis clarified that; type of feeding did not consider a significant risk factor for maintenance of parasitic infection among dogs (p>0.05) and that undercooked feed was 7.62 times carrying the risk of enteric parasitic infection than cooked and dry feed (Table-3). These results were in accordance with those reported by Zelalem and Mekonnen [33] and Ahmed *et al*. [11]. Uncooked feed that were introduced to dog in Egypt mainly included viscera of fish and chicken that may carry many parasitic infection, while cooking of feed can kill or inactivate infective eggs or cysts of gastrointestinal helminthes which could be transferred to dogs via feed [33].

Communal housing of dogs was found to be a significant risk factor for acquiring enteric parasitic infection than individual housing (42.7% vs. 12.7%, COR=5.10, 95% CI=2.04-12.75, p<0.000). This may be related to direct spread of infection through feeds contaminated with faces of dogs infected with various parasitic infections.

Univariate logistic regression revealed that nomadic dogs were 67.36% more likely to be infected with enteric parasites than military dogs (95% CI=8.09-560.8, p<0.000) followed in descending order by dogs from rural areas (COR=26, 95% CI=3.14-215.54, p=0.003) and dogs from high standard areas (COR=11.87, 95% CI=1.37-102.69, p=0.025). Mateus *et al*. [36] reported differences in the prevalence of enteric parasites according to nature of the examined dogs (59.8% in environmental dogs, 57.44% in farm dogs and 81.18% in hunting dogs) in Portugal. Higher frequency of enteric parasitic infection in nomadic dogs may be related to the free roaming nature of these dogs that categorize them as stray dogs. These dogs roam freely and mostly live outdoors, eat garbage, scavenge rodents and other dead animals that may be a potential source for their infection with enteric parasites. Furthermore, high infection rate in domiciled dogs from rural areas than high standard areas may be related to lower hygienic measures, communal nature of living, less or no veterinary attention in contrast to better hygienic and veterinary care and keeping majority of dogs in flats in urban areas [37]. Ahmed *et al*. [11] in Egypt recorded parasitic infection in 7.5% of police dogs compared to 40% in house dogs. In Turkey, Senlik *et al*. [23] found that 30.4% of Turkish military dogs frequently harbor intestinal nematodes. They concluded that, however, the hygienic measures, regular deworming, high quality feeding of police and house dogs, different parasites were recorded.

### Occurrence of enteric parasitic infection in humans

In developing countries, *Cryptosporidium* spp. infections have been frequently incriminated in diarrheal disease in humans, particularly in children younger than 5 years. *Cryptosporidium parvum* (human and cattle genotype) and *Cryptosporidium hominis* have been identified in a majority of human patients. Other species (*Cryptosporidium meleagridis* and *Cryptosporidium felis*) and genotypes (*C. parvum* dog genotype) were diagnosed in a proportion of immunocompetent children [38]. In this study, *Cryptosporidium* spp. was the most frequently detected parasites in human stool samples (9.33%). In Egypt, studies on Cryptosporidium spp. among individuals with diarrhea attending inpatient and outpatient clinics reported prevalence ranges from 0% to 49% [26,39-41]. Sargent *et al*. [42] detected *Cryptosporidium* spp. in dogs and cats and concluded that these animals may represent potential sources of infection for human. Moreover, the possibility of *Cryptosporidium* transmission among human and dogs has been reported by Xiao *et al*. [43] who diagnosed *Ctenocephalides canis* infection in 2 children and their dog during the same period.

*A. lumbercoides* was the second most frequently detected enteric parasites in human stool (3.33%). Similar prevalence of *A. lumbercoides* in stool samples (4.9%) was previously recorded in Egypt by Esteban *et al*. [44]. Higher frequencies were recorded by El Sahn *et al*. [45] in Egypt, Dreyer *et al*. [46] in Brazil and Agi [47] in Nigeria. Other studies showed lower prevalence that were 1.4% [48] and 1.8% [26]. Shalaby *et al*. [49] detected A. lumbricoides in 8% of examined dogs and clarified the role played by dogs as a reservoir for and an environmental contaminator with A. lumbricoides which in turn increasing the risk of humans infection.

*Ancylostoma* spp. and *Heterophyes* spp. were the third detected enteric parasites in human stools (2.67%, each). *Ancylostoma* spp. has been considered one of the most frequent intestinal parasites of dogs [6]. Not only *A. caninum* but also different *Ancylostoma* species are involved in human infection mainly cutaneous larva migrans [50]. Eosinophilic enteritis and unexplained abdominal pain with peripheral eosinophilia are other manifestation of A. caninum infection in humans. Bahgat *et al*. [51] detected IgG antibodies to A. caninum in 11 out of 95 (11.6%) patients with obscure acute or recurrent abdominal pain.

Heterophyiasis is a highly endemic disease in Egypt. Fishermen (33.8%) [52] and local residents in northern Egypt (13.3%) [53] are the highest susceptible to infection. Detection of *Heterophyes* spp. in fecal samples of dogs (3.85%) and stool of humans (2.67%) in this study substantiates the evidence that they were infected through a common source of infection that may be fish. *Heterophyid* infection has been reported in 22% of brackish water fish and 42% for fresh water fish with an overall prevalence of 32% [53]. However, Ibrahim and Soliman [54] detected *heterophyid* metacercaria in 95.4% of fresh water fish examined in Ismailia province. Moreover, H. heterophyes was detected in 3% of stray cats in Kafr Elsheikh province in the northern region of the Delta [55] which may be scavenged by dogs or contaminates human food especially green vegetables and in turn transmit the infection to dogs and man.

*H. nana* and *Paragonimus westermani* were the fourth prevalent enteric parasites in human stools (1.33%, each). *H. nana* was recorded by Merwad [56], Esteban *et al*. [44], El Shazly *et al*. [26], and Bakr *et al*. [57] in stool samples in Egypt with percentages of 6.7, 4.9, 3.9, and 3, respectively. Moreover, *H. nana* frequency varied from country to country; 7.2% in Morocco [58], 14.29% in Venezuela [59], 0.1% in Libya [60] and 11.3% in Ecuador [61]. *H. nana* infection may be either asymptomatic or symptomatic and mainly affect preschool and primary schoolchildren. Symptomatic manifestations include; abdominal pain, nausea, vomiting, and diarrhea, anorexia, itching, irritability, sleeplessness and enuresis [62], as well as anemia due to reducing the intestinal absorption of vitamin B12 and folic acid [63].

“Paragonimiasis is a zoonotic disease in which humans may act as definitive hosts. The prevalence of paragonimiasis throughout the world is difficult to ascertain. It was estimated 293 million people are at risk, whereas several million are actually infected with paragonimiasis” [64]. The acute phase (invasion and migration) of paragonimiasis is manifested by diarrhea, abdominal pain, fever, cough, urticaria, hepatosplenomegaly, pulmonary abnormalities, and eosinophilia. Pulmonary manifestations include cough, expectoration of discolored sputum, hemoptysis, and chest radiographic abnormalities are the main characteristics of the chronic phase. More severe manifestations occur as a result of extrapulmonary localization of the adult worms, especially when the brain is involved [65]. Detection of *Paragonimus* spp. eggs in 0.77% of dogs and 1.33% of human in this study may be related to a common source of infection. This source may be raw or undercooked crustaceans.

In human, examining stool for parasite and eggs using the direct parasitological diagnosis is not useful and extremely difficult because *T. canis* cannot mature to the adult stage, thus serological methods are the diagnostic method of choice. Serological diagnosis of toxocariasis can be done using ELISA for detection of anti-*T. canis* antibodies against larval excretory secretory antigen [66].

The frequency of anti-*T. canis* antibodies among human populations in Egypt in this study (24%, 36/150) were higher than those recorded in developed countries; 0.7% in New Zealand, 1.6% in Japan, 2.4% in Denmark, 3.9% in Canada, 7.5% in Australia, 14% in USA, 15% in Poland [66-68]. By contrast, higher seroprevalence have been recorded in less developed countries in; Asia (81% in Nepal, 63.2% in Indonesia and 58% in Malaysia), Africa (30% in Nigeria, 45% in Swaziland and 93% in La Reunion), and South America (36% in Brazil and 37% in Peru) [69-71].

### Occurrence of *Toxocara* IgG antibodies in humans and risk factor assessment

The frequency of anti-*T. canis* antibodies among the examined males (26.66%) was higher than females (20%). These results agreed with Won *et al*. [72], Roldan *et al*. [69] and Sariego *et al*. [7]. “High frequency in males than females may be attributed to the observation that males tend to spend more time outdoors than females and are generally less particular about personal hygiene, thus being at risk of contact with parasites” [67]. On the other hand, Alonso *et al*. [73] and Alderete *et al*. [74] reported that the association between male gender and seropositivity to *toxocara* infection is not consistently present. Regarding gender as a risk factors for acquiring enteric parasitic infection, univariate regression analysis verified that gender did not seem to be an important risk factor attributed to *T. canis* infection among humans in Egypt (COR=1.46, 95% CI=0.66-3.19, p=0.35). This is in accordance with the results reported by Fan *et al*. [75] in Taiwan, Ajayi *et al*. [76] in Nigeria, and Sadjjadi *et al*. [77] in Spain.

Moreover, age group (7-15 years old) was found to be significantly infected with enteric parasitic infection more than other age groups (COR=6.93, 95% CI=1.75-27.43, p=0.006). Fan *et al*. [75] stated that the age was not an important factor related to *T. canis* infection.

On the other hand, a non-significant association was observed between raising of dogs and *toxocara* infection in humans in Egypt. A recent study indicated that dogs infected with *T. canis* might infect people by direct contact because of the high density of embryonated eggs in their fur [78]. Nevertheless, high seropositive rates of 29.85% in dog owners and 19.27% in non-owners of dogs in the present study suggest that these two groups are equally at risk of being infected. The results are in line with those of Fan *et al*. [75] who reported a similar frequency of anti-*T. canis* antibodies among dog owners (79.4%) and non-owners (67.9%). Moreover, Woodruff *et al*. [79] observed that 50% of patients with clinical toxocariasis had never owned a dog or had close contact with pets.

A non-significant association were found between playing with soil (COR=1.33, 95% CI=0.50-3.57, p=0.567), eating of raw vegetables (COR=1.35, 95% CI=0.53-3.42, p=0.528) and lacking of hand washing before meals (COR=0.73, 59% CI=0.30-1.78, p=0.49) with the frequency of anti-*T. canis* antibodies in the examined human sera. Fu *et al*. [80] also recorded insignificant association between contact with soil or consumption of raw vegetables with the frequency of anti-*T. canis* antibodies in human sera. In spite of insignificant association between eating of raw vegetables and the seropositivity to *T. canis* infection, raw vegetable should be considered as a potential source of *T. canis* since its detection in different types of vegetables all over the world Kozan *et al*. [81] in Turkey, Abougraina *et al*. [82] in Libya and Doaa [83] in Alexandria, Egypt. Doaa [83] detected *T. canis* eggs in rocket (25%), lettuce (31.7%), parsley (20%), leek (8.3%) and green onion (10%) with a total contamination of 19%.

Although there was no significant association between contact with soil and seropositivity to *T. canis* infection, contact with soil should be done with precaution and proper personal hygiene must be carried out after contact with soil since the reported existence of *Toxocara* eggs in soil samples in previous studies; Rinaldi *et al*. [84] in Italy, Martinez-Moreno *et al*. [14] in Spain, Mizgajska *et al*. [85] in Poland and de Castro *et al*. [86] in Brazil.

In Egypt, there is no information about whether the frequency of anti-*T. canis* antibodies in various occupational groups either exposed or not to dogs. The present study was performed to investigate whether the frequency of anti-*T. canis* antibodies was associated with certain occupation. Students were the most frequently infected groups (34.29%), followed by nomadic people (32%), house guarders (28%), and house wives (20%), however, military workers (13%) and employees (10) were the least infected groups. Univariate regression analysis revealed no significant association between occupation of the examined persons and the frequency of anti-*T. canis* antibodies. In Egypt, Sabry and Lotfy [25] detected anti-*T. canis* antibodies in 3/20 (15%) of workers with no history of contact with dogs and 0% in those contacted with dogs. Sadjjadi *et al*. [77] in Iran detected IgG against *T. canis* in 25.6% of the examined children. Anti-*T. canis* antibodies were detected in 1.8% of gardeners and 13% of waste pickers in Mexico [87,88]. Fan *et al*. [68] stated that young children up to the age of 12 years appear to be the primary population susceptible to *T. canis* infection because of dirt pica, poor hygiene or frequent contact with dirt.

## Conclusion

Detection of enteric parasites in dogs and human in Egypt substantiate the role posed by dogs in transmitting zoonotic parasites to humans and knock an alarm for common sources of infection for humans and dogs. Common sources may be infected fish or contaminated vegetables that are consumed by dogs or humans or even infected rodents that may contaminate their feed. This pilot study necessitate the need for similar studies and tracing such infection in fish, vegetables, rodent that may be responsible for infecting humans and dog in order to understand the epidemiology of zoonotic parasitic infection transmitted from dogs to human.

## Authors’ Contributions

MAIA and LMAS: Planned the study design, collected and examined samples, drafted and revised the manuscript. Both authors read and approved the final manuscript.
